# A Review of Respiratory Biologic Agents in Severe Asthma

**DOI:** 10.7759/cureus.5690

**Published:** 2019-09-18

**Authors:** Nathaniel Johnson, Blessy Varughese, Marianne A De La Torre, Salim R Surani, George Udeani

**Affiliations:** 1 Pharmacy, Corpus Christi Medical Center, Corpus Christi, USA; 2 Internal Medicine, Corpus Christi Medical Center, Corpus Christi, USA; 3 Internal Medicine, Texas A&M Health Science Center, Temple, USA; 4 Miscellaneous, Corpus Christi Cancer Center, Corpus Christi, USA

**Keywords:** severe asthma, exacerbations, ige, respiratory biologics, antibody, t-helper cells, forced expiratory volume in 1 second (fev1)

## Abstract

Asthma is a common but complex chronic inflammatory heterogeneous lung disease, punctuated by the pathophysiological phenomenon of airway narrowing, coupled with symptoms of wheezing and coughing. The mechanism behind these symptoms is due to migration of eosinophils, mast cells, and CD4 T-helper cells into the submucosa of the airway, leading to hyperresponsiveness to common allergens, microorganisms, oxidants, pollutants, and consequently, airway remodeling. There is evidence that this migration is mediated by inflammatory cytokines derived from T-helper 2 (Th2) cells and type 2 innate lymphoid cells (ILC2), such as interleukins 4, 5, and 13. These cytokines lead to an increase in immunoglobulin E (IgE) production. Additionally, thymic stromal lymphopoietin (TSLP) released from airway epithelium can activate Th2 cells, innate lymphoid cells, or both. All have proven significant in the promotion of chronic airway inflammation and remodeling. In the past, most treatment strategies for this condition focused on two drug classes: β2 agonists (both short- and long-acting), and inhaled corticosteroids. Other treatments have included maintenance drugs, such as leukotriene receptor antagonists, long-acting anticholinergic agents, and theophylline. None of these, however, directly impact the interleukin or IgE pathways in a meaningful manner. Clinical trials of novel agents impacting these pathways have demonstrated efficacy and improved outcomes in asthma exacerbations, control, and forced expiratory volume in 1 second (FEV_1_) in patients with severe asthma. Future treatments in asthma will focus on drugs that target these aforementioned cytokines.

## Introduction and background

Asthma is a significant economic burden in the United States (US), based on morbidity, mortality, treatment, and lost productivity due to absenteeism from work and school. Nurmagambetov et al. examined data from 2008 - 2013 and found that the cost of asthma medical treatments alone was $3,266 per individual (in 2015 inflation-adjusted US dollars) [1]. Broken down further, this amounted to approximately $1,830 from prescription therapies, $640 from in-office visits, $105 in emergency room visits, $529 in admissions due to exacerbations, and $176 in post-discharge outpatient visits. During the five-year study period, asthma was implicated in $3 billion in losses due to absenteeism from work and school, $29 billion due to costs for asthma-related mortality, and $50.3 billion in medical treatment costs. Based on pooled sample data, the overall combined cost of asthma in the US was estimated at $81.9 billion for the 2013 calendar year.

Asthma is typically managed using both pharmacological and non-pharmacological approaches. Allergen avoidance has been the main focus of the non-pharmacological approach. Pharmacological treatments have included β2 agonists, inhaled corticosteroids, leukotriene receptor antagonists, long-acting anticholinergic agents, and theophylline. Most patients respond to these treatments, but a certain subset experiences severe asthma, which is refractory (even to higher dosages) of these regimens. Research has continued in the deployment of novel asthma treatments, focusing on cytokine pathways when developing therapeutic targets for the management of such severe asthma. This paper will focus on the cytokines that have been implicated in severe asthma, currently targeted for potential novel therapeutic agents. These include T-helper 2 (Th2), type 2 innate lymphoid cells (ILC2), interleukin 4 receptor alpha (IL-4Rα), IL-4, IL-5, IL-13, thymic stromal lymphopoietin (TSLP), and non-Th2 pathways.

Interleukins 4, 5, and 13 (derived from innate lymphoid cells and T-helper cells), as well as immunoglobulin type E (IgE), have become major targets for therapeutics in recent years for the roles they play in immune response and allergic pathogenesis [2]. Studies of cytokine inhibitors (anti-interleukin-5, anti-interleukin-4Rα, and anti-interleukin-13) in asthmatic patients with recurrent exacerbations and high concentrations of eosinophils, despite the use of inhaled corticosteroids, have reported positive outcomes in terms of exacerbation frequency, symptom control, and forced expiratory volume in 1 second (FEV1) [3-6]. Unfortunately, these agents are quite expensive and are usually reserved as an add-on therapy for patients who have proven refractory to the maximum dosage regimen using the current standard-of-treatment medications, such as inhaled corticosteroids (ICS) and long-acting β2 agonists (LABAs). However, this idea is changing with emerging new literature and research.

Asthmatic patients with allergic-type asthma have notably higher circulating levels of IgE compared to the general population [7-8]. Sensitization to common allergens, such as pet dander, mold, insects, and pollen, can result in the formation of IgE specific to the allergen. Further exposure produces an immune response and classic asthma symptoms of wheezing, coughing, and airway obstruction [9-12]. Attenuation of this response is a primary objective of acute asthma exacerbations, while the reduction in the severity and number of exacerbations is a crucial goal in maintenance treatment [13]. The ability to inhibit the immune response and reduce the severity and number of exacerbations in severe patients with allergic-type asthma, through the use of monoclonal antibodies, is a valuable addition to the clinician’s toolbox.

This review will focus on the following drugs: omalizumab (Xolair®, Novartis Pharmaceuticals Corp., E. Hanover, NJ, USA), reslizumab (Cinqair® - US, Teva Pharmaceuticals USA, Inc., North Wales, PA); Cinqaero® - EU, Teva Pharmaceuticals Ltd., Petach Tikva, Israel), mepolizumab (Nucala®, GlaxoSmithKline, Warren, NJ), benralizumab (Faserna®, AstraZeneca, Cambridge, United Kingdom), and dupilumab. As seen in Table 1 (see Appendix), we will review the mechanisms of action, in vitro activity, pharmacodynamics, and pharmacokinetics of these drugs. This paper will also present key clinical trial data found in Table 2 (see Appendix).

## Review

Omalizumab

Omalizumab (Xolair®, Novartis Pharmaceuticals Corp., E. Hanover, NJ) is a recombinant humanized deoxyribonucleic acid (DNA)-derived IgG1 monoclonal antibody. Currently, it is the only anti-IgE agent available for use in asthma approved in the US for use in patients who display all of the following characteristics: at least six years of age, have a diagnosis of moderate to severe persistent asthma, achieve inadequate control of asthma symptoms despite the use of medium- or high-dose inhaled glucocorticoids, have a serum IgE level between 30 and 700 international units per milliliter (IU/mL), and are sensitive (as demonstrated by a positive skin test) to specific allergens year-round [14].

The US Federal Drug Administration (FDA) clinical trials demonstrated a lower frequency of exacerbations in asthmatic patients with omalizumab, as well as a decrease in overall corticosteroid exposure. However, it is not appropriate for treatment of acute symptoms. Corticosteroids should not be discontinued abruptly upon initiation of therapy but should instead be carefully titrated down as the patient responds to therapy. The clinician should also monitor for complications, such as eosinophilia, vasculitic rash, worsening pulmonary function, cardiac dysfunction, and neuropathic pain, particularly following any reduction in corticosteroid use [3].

Omalizumab ultimately prevents activation of basophils and mast cells. This drug binds with high affinity to the heavy chain C-epsilon-3 (C-ε-3) domain of IgE, which in turn binds to the FcεRI receptors on basophils and mast cells. When sufficiently low amounts of circulating IgE are achieved, the activation of early- and late-phase allergic response is attenuated, causing a disruption of the allergic cascade, reducing eosinophil-mediated inflammation, and preventing mast cell degranulation. In addition, the expression of FcεRI is down-regulated once the omalizumab therapy is underway. The time frame in which down-regulation occurs appears to differ between basophils and mast cells, but mast cells are the rate-limiting step. Lin et al. found that omalizumab patients showed a 90% mean decrease in FcεRI expression after 70 days but virtually no decrease within the first seven days [4]. In contrast, another study of omalizumab in patients with ragweed-induced allergic rhinitis demonstrated a 73% reduction in FcεRI expression within the first seven days of treatment [5]. Therefore, it appears that using omalizumab to control severe, refractory asthma does not demonstrate its full benefit as quickly as when used for other indications.

Omalizumab is highly specific to IgE and will not bind to other immune globulins (such as IgG and IgA) nor can it bind FcεRI itself; this prevents not only inappropriate immune suppression for other functions but also activation of an asthmatic immune response by the drug. However, if IgE is already attached to FcεRI, it cannot displace it; thus, omalizumab is not suitable for use in the acute treatment of asthma exacerbation. Unfortunately, there is a very low threshold number of IgE molecules needed for immune activation (~2,000), and a vast number of available sites at which it can bind (estimates range from 10,000 to 1,000,000). This means that an extremely high affinity for the IgE molecule and subsequent binding of nearly all circulating IgE is required for omalizumab’s clinical efficacy [5].

The US Food and Drug Administration (FDA) clinical trials demonstrated a lower frequency of exacerbations in asthmatic patients with omalizumab, as well as a decrease in overall corticosteroid exposure, but it is not meant for the treatment of either acute bronchospasm or status asthmaticus. Dosing is based on body weight and pretreatment serum IgE levels. Dosing adjustments can be made for significant changes in body weight; however, it should not be adjusted for IgE levels taken either during the course of treatment, up to one-year post-treatment, or during an interruption in therapy. Past the one-year mark, IgE levels are considered to be at baseline and may be used to determine dosing once again if therapy resumes. No dosage adjustments are provided in the manufacturer’s labeling for either renal or hepatic impairment. However, it is recommended that doses exceeding 150 mg be distributed over more than one injection site [14].

Although the FDA only approved its use in persistent asthma and failure of inhaled corticosteroid, the 2018 Global Initiative for Asthma (GINA) guidelines recommended that omalizumab only be considered for adjunctive therapy in patients with uncontrolled moderate or severe allergic asthma, despite the concomitant use of a medium-dose or high-dose inhaled corticosteroid (ICS) and a long-acting β2-agonist (LABA) [13].

While increases in rates of malignancy have been reported in short-term studies of omalizumab, it remains to be seen whether these rates are increased over the long-term. The clinician is encouraged to consider the risk/benefit ratio for their patient when deciding whether to use any monoclonal antibody therapy. Omalizumab has been explored through several randomized, placebo-controlled clinical trials as an add-on treatment for children and adults with allergic asthma. Three such trials are described below:

Omalizumab Trial I

Trial I was a prospective, multicenter, randomized, parallel-group, double-blind, placebo-controlled trial conducted in 2011 to evaluate the efficacy and safety of omalizumab in patients with inadequately controlled severe asthma using high-dose ICS (equivalent to ≥ 500 mcg of fluticasone twice daily) and LABAs, either with or without additional controller therapy [15]. The study analyzed 850 patients, 12 to 75 years of age, and accounted for baseline controller use through further stratification into three groups: M1, M2, and M3. M1 consisted of ICS, plus LABA alone, M2 was ICS, plus LABA, plus ≥ 1 additional controller medication (but not oral corticosteroids (OCS)), and M3 was ICS, plus LABAs, plus OCS.

The primary endpoint was the number of asthma exacerbations experienced over the 48-week study period, requiring an increase in an average daily dose of ≥ 20 mg of prednisone or equivalent OCS dose adjustment [15]. The exacerbation rate was significantly lower in the omalizumab group than in the placebo group, amounting to a 25% reduction in relative exacerbation rates (0.66 vs. 0.88; p = 0.006). Secondary endpoints were analyzed by mixed-effects models and included changes from baseline to Week 48 in the mean daily number of puffs of albuterol, mean total asthma symptom score, and mean overall score on the standardized version of the Asthma Quality of Life Questionnaire (AQLQ). Omalizumab demonstrated greater increases in the AQLQ, decreased mean albuterol puffs/day, and reductions in mean asthma symptom scores compared with placebo, as well as a greater proportion of patients who improved from baseline to Week 48 greater than the minimal clinically important difference (67.8% vs. 61.0%; p = 0.042). Unfortunately, the study was stymied by a low rate of retention amongst the study patients, both in the treatment and placebo groups, with 80.6% of the omalizumab group and 77.8% of the placebo group completing the study.

*Omalizumab* *Trial II*

This 24-week trial analyzed the effect of omalizumab in patients with persistent allergic asthma, using the National Heart, Lung, and Blood Institute (NHLBI) Asthma Control Guideline, Step 4 or above, as their control therapy (medium-dose ICS, plus LABA, or medium-dose ICS, plus a leukotriene receptor antagonist, theophylline, or zileuton) [16]. The primary efficacy endpoint was the change in Asthma Control Test (ACT) score, with omalizumab demonstrating a non-significant increase in the least-squares means (LSMs) over placebo (5.01 and 4.36, respectively, p = 0.1779). The secondary efficacy endpoint was the Investigator’s Global Evaluation of Treatment Effectiveness (IGETE) score; more patients in the omalizumab group had IGETE scores in the “excellent” range vs. placebo, but again, the difference was not significant (20% vs. 15%, p = 0.1177). The only significant differences observed for omalizumab were changes in the ACT scores (LSMs: 6.66 vs. 5.27, p = 0.0334) and IGETE (p = 0.0321) at Week 24 in a specific subgroup of patients consisting of those with very poorly controlled asthma, defined as an ACT score ≤ 15 at baseline. No significant differences were found in serious adverse drug events or deaths. The study concluded that omalizumab consistently improved asthma control, but the difference compared to placebo was only detected in specific subgroups of patients.

*Omalizumab* *Trial III*

This trial was a double-blinded, placebo-controlled, multicenter, parallel-group trial designed to evaluate the efficacy and safety of omalizumab in ICS controller-dependent asthma patients, defined as requiring 420 to 840 mcg/day of beclomethasone dipropionate (BDP) or an equivalent ICS dose for ≥ 3 months prior to randomization [17]. Those not using BDP were changed to an equivalent dose of BDP during the four to six-week run-in period, adjusting the dose to achieve adequate symptom control for a four-month period, called the stable steroid phase. Subsequently, doses of BDP were tapered down by 25% of the baseline dose every two weeks for eight weeks or until discontinuation or worsening of asthma symptoms were noted. A mean total symptom score of 3 or more (range: 0 - 9), over the last two weeks of the run-in period, was required for the patient to be eligible for randomization to omalizumab or placebo groups.

The primary endpoint was the number of exacerbation episodes experienced by a patient during the steroid reduction period and during the stable steroid phase. One or more exacerbations during the 28-week study period were experienced by 14.6% of the omalizumab patients and 23.3% of the placebo patients (p = 0.009). In addition, the mean number of per-subject exacerbations (0.28 vs. 0.54; p = 0.006) and mean duration of exacerbations (7.8 vs 12.7 days; p < 0.001) were also lower with omalizumab than with the placebo. More omalizumab recipients than placebo recipients achieved at least a 50% reduction in the BDP dose (72.4% vs 54.9%; p < 0.001). Secondary variables measured during the study included the number of patients experiencing at least one exacerbation, daily asthma symptoms, rescue medication use, and pulmonary function. For these, omalizumab improved daily asthma scores vs. the placebo after four weeks of treatment, and rescue medication use was significantly reduced vs. the placebo for most of the measured weekly intervals. Increases in morning peak expiratory flow (PEF) were greater in the omalizumab group than in the placebo group from the baseline to the end of the study period. The mean change from baseline PEF in the omalizumab group was 18.5 L/min, while the placebo group had a mean change of 6.9 L/min. Mean predicted FEV_1_ increased from 68.2% to 72.53% in the omalizumab group, and from 67.7% to 69.1% in the placebo group. These improvements in FEV_1_ were statistically significant and remained consistent throughout the entire study (p values < 0.001 to 0.019). The frequency of adverse effects in the two groups were not significantly different, with 89.2% of omalizumab patients and 89.1% of placebo patients reporting at least one adverse effect. The study concluded that omalizumab was safe and effective for patients with severe allergic asthma exhibiting poor control or frequent exacerbations despite appropriate therapy [17].

Omalizumab’s pharmacokinetic and pharmacodynamic information [14] is found in the Appendix, Table 1.

Reslizumab

Reslizumab (Cinqair® - US, Teva Pharmaceuticals USA, Inc., North Wales PA, USA; Cinqaero® - EU, Teva Pharmaceuticals Ltd., Petach Tikva, Israel) is an immune globulin G4-kappa-type (IgG4 κ) monoclonal antibody, approved by the FDA in 2016 as an add-on maintenance therapy in individuals 18 years of age and older with eosinophilic-type asthma. This agent was initially developed by several companies, including Schering-Plough [18]. The agent predominantly binds human interleukin 5 (IL-5), a major cytokine associated with differentiation, maturation, recruitment, and activation of human eosinophils. Eosinophils play a significant role in airway inflammation, and eosinophilic-type asthma is a phenotype associated with elevated levels of eosinophils in the lungs and sputum. There is a positive correlation between the numbers of eosinophils in the blood and bronchial fluid and the severity of the asthma symptoms.

Reslizumab impairs the bioactivity of IL-5 by blocking its binding ability to the IL-5R α-chain complex expressed on the surface of eosinophils, thereby inhibiting IL-5 signaling and reducing both the production and survival of eosinophils [19-20]. The recommended dosage for reslizumab is 3 mg/kg administered once every four weeks via intravenous infusion over a 20 - 50 minute period. The infusion duration depends on the total infusion volume and patient’s weight. Reslizumab is available as 100 mg/10 mL (10 mg/mL) and must be diluted in a 50 mL bag of 0.9% sodium chloride prior to administration. The recommended drug administration set is an in-line, low protein-binding filter that has a pore size of 0.2 microns [20].

Reslizumab has demonstrated notable inhibition of eosinophilia in a variety of animal models, including skin, rabbits, monkeys, and pigs [21-24]. Characteristics of the agent were similar across the spectrum of healthy individuals, patients with asthma, and other populations, with inter-individual variability in peak and trough levels in the range of 20 - 30% for both children and adults. Peak serum levels were observed at the end of infusion and declined via a biphasic approach. Reslizumab plasma concentrations were dose-dependent and accumulated in the range of 1.5 to 1.9-fold after multiple-dose administrations [20].

The distribution volume of reslizumab is 5 L, with a clearance of about 7 ml/hour, and elimination half-life of 25 to 30 days. Following a dose of intravenous (IV) 1 mg/kg in patients with asthma, the following plasma concentration pharmacokinetic parameters were observed: mean maximal plasma concentration - 30.3 mcg/ml within 6.9 hours post-dose, declining to 0.87 mcg/ml by day 90 and subsequently to 0.43 mcg/ml by day 120 [25]. Enzymatic proteolysis degrades reslizumab into small peptides and amino acids, similar to other monoclonal antibodies [26]. In vitro data indicate that it is unlikely for IL-5 and reslizumab to affect cytochrome P450 1A2, 2B6, or 3A4 enzyme activity. Mild hepatic impairment, mild to moderate renal impairment, or the concomitant use of leukotriene antagonists or corticosteroids does not significantly affect the pharmacokinetic profile of reslizumab [20].

Clinical trials leading to the approval of reslizumab included four double-blind, randomized, placebo-controlled studies (Studies I-IV).

Reslizumab Studies I/II

Nine hundred and fifty-three patients were enrolled in the reslizumab Studies I and II, with eosinophilic asthma and eosinophil counts of at least 400/mcL and at least one asthma exacerbation which required corticosteroid therapy over a 12-month period. These were simultaneous studies which lasted 52 weeks, the objective of which was to determine the efficacy and safety of reslizumab in decreasing asthma exacerbations. Patients were treated with IV reslizumab, 3 mg/kg, or placebo every four weeks for a total of 13 doses. Oral corticosteroid maintenance therapy, up to an equivalent dose of prednisone, 10 mg, were allowed during the studies. Most patients (82%) were managed with a medium- to a high-dose regimen of an inhaled corticosteroid and a long-acting β agonist (ICS/LABA) at baseline [20, 27].

The primary endpoint of both studies was the frequency of asthma exacerbations, further defined as a deterioration of asthma based on one of two criteria. The initial criterion was the need for and use of systemic corticosteroids or augmented use of inhaled corticosteroids for three or more days. A twofold increase in corticosteroid use for three or more days was considered an exacerbation of asthma in individuals already taking corticosteroid treatment. The second criterion observed the frequency of emergency management, described as an impromptu visit to an emergency department, a health care provider for nebulizer treatment, or hospitalization associated with asthma [27].

Patients in the reslizumab group had lower rates of total asthma exacerbations compared to those in the placebo group, with observed relative reductions of 50% in Study I, and 41% in Study II. Patients in the reslizumab group had fewer exacerbations necessitating systemic corticosteroid use compared to those in the placebo group, with relative reductions of 45% and 39% observed in Studies I and II, respectively. Relative reductions of 66% and 69% in Studies I and II, respectively, were observed in the reslizumab group (when compared with the placebo group) for exacerbations requiring hospitalizations or emergency department visits [27].

Reslizumab Study III

​​​​​​​Study III consisted of 315 eosinophilic asthma patients with eosinophil counts of at least 400/mcL, divided into three groups. The study was designed to compare the efficacy of reslizumab (3 mg/kg and 0.3 mg/kg to placebo in lung function improvement) in eosinophilic asthma patients. Patients requiring oral corticosteroids as maintenance therapy were excluded. The primary endpoint was the measured change in FEV_1_ during a 16-week period. Baseline FEV_1_ measurements were obtained at four, eight, 12, and 16 weeks. The mean of the FEV_1_ measurements was estimated via the mixed-effect model for repeated measurements, with a positive variation indicative of improved asthma control. There were two reslizumab groups and one placebo group in Study III. One of the reslizumab groups of patients were treated with 3 mg/kg IV every four weeks (a total of four doses) over a 16-week period. The second group of reslizumab patients received 0.3 mg/kg of the drug IV every four weeks (a total of four doses) over a 16-week period. The patients in Group 3 (placebo) were administered IV placebo every four weeks (a total of four doses) over a 16-week period [20].

Compared to baseline, FEV_1_ improvements of 0.286 L and 0.242 L were observed in the reslizumab (3 mg/kg and 0.3 mg/kg) groups, respectively, whereas FEV_1_ improved by 0.126 L in the placebo group after 16 weeks. The average changes in FEV_1_ were compared with the placebo and each of the reslizumab study groups. In the reslizumab 3 mg/kg group, an FEV_1_ change of 0.160 L greater than placebo (95% confidence interval (CI), 0.060 - 0.258; p = 0.0018) was observed. In the reslizumab 0.3 mg/kg group, an FEV_1_ change 0.116 L greater than placebo (95% CI, 0.016 - 0.215; p = 0.0237) was observed [20].

Reslizumab​​​​​​​ ​​​​​​​Study IV

The objective of Study IV was to determine the efficacy of reslizumab, 3 mg/kg, towards improving pulmonary function in 497 patients with moderate-to-severe eosinophilic asthma. Individuals on maintenance oral corticosteroid therapy were excluded from this study; however, inclusion was not based on eosinophil counts. Approximately 80% of the patients had eosinophil counts less than 400/mcL. Reslizumab, 3 mg/kg IV, was administered every four weeks (a total of four doses) over a 16 week period. The initial primary endpoint measured effectiveness via FEV_1_ variations over a 16 week period, and the reslizumab 3 mg/kg group had an average FEV_1_ increase of 0.076 L greater than the placebo group (95% CI, -0.006 to 0.158; p = 0.076). An updated and current primary outcome measure assessed FEV_1_, determined in liters, in comparison to baseline eosinophil count, measured in 109/L. In the reslizumab 3 mg/kg group, a change of 0.0229 FEV_1_ L/eosinophil x 109/L was observed, compared to an FEV_1_ change of −0.2778 L/eosinophil 109/L in the placebo group [28].

Reslizumab’s pharmacokinetic and pharmacodynamic information [29] is found in the Appendix, Table 1.

Mepolizumab

Mepolizumab (Nucala®, GlaxoSmithKline, Warren, NJ, USA) is an IgG1 κ monoclonal antibody, FDA-approved for use as an add-on maintenance treatment in patients 12 years or older with severe eosinophilic-type asthma who experienced recurrent exacerbations despite being on inhaled corticosteroid therapy, with or without the addition of oral corticosteroids. Through the addition of mepolizumab, asthma control appeared to be better obtained without the need for long-term steroid use [30-31].

Similar to reslizumab, mepolizumab impairs the bioactivity of IL-5 by blocking its binding ability to the IL-5 α-chain complex, inhibiting eosinophil production, and survival. Dosing for mepolizumab is a single 100 mg subcutaneous injection into the upper arm, thigh, or abdomen every four weeks. No dose adjustments are recommended for geriatric patients, pediatric patients (> 12 years old), or those with renal or hepatic impairment [32]. It is not indicated as a relief for acute bronchospasm, status asthmaticus, or other eosinophilic conditions, and patients should not discontinue their previous oral or inhaled corticosteroids abruptly after the start of mepolizumab use [30]. However, a gradual decrease in corticosteroid therapy may be initiated if appropriate [32].

Hypersensitivity reactions, such as angioedema, bronchospasm, hypotension, urticaria, and rash, have been reported with mepolizumab, often requiring discontinuation. In patients with preexisting helminth infection, treatment must be completed before starting mepolizumab therapy. If a helminth infection occurs during mepolizumab therapy and the patient experiences difficulty with anti-helminth treatment, discontinuation of mepolizumab therapy is recommended until the parasitic infection is resolved. Additionally, case reports of herpes zoster infections have been noted; thus, it is recommended that patients beginning therapy receive vaccination against varicella if medically appropriate [32]. Other common adverse events reported are headaches, injection site reactions, back pain, and fatigue [33].

In 2015, the FDA approved mepolizumab as add-on maintenance therapy through the evaluations of three clinical trials (Dose Ranging Efficacy and Safety with Mepolizumab in Severe Asthma (DREAM) [33], Mepolizumab as Adjunctive Therapy in Patients with Severe Asthma (MENSA) [31], and Mepolizumab Steroid-sparing Study in Subjects with Severe Refractory Asthma (SIRIUS) [34]). One additional clinical study, A Multi-centre, Open-label, Long-term Safety Study of Mepolizumab in Asthmatic Subjects Who Participated in the MEA115588 or MEA115575 Trials (COSMOS), was performed to evaluate the long-term efficacy and safety of mepolizumab utilizing patients from within the MENSA and SIRIUS trials [35]. With each trial conducted, data demonstrated a reduction in the numbers and severity of asthma exacerbations, improved quality of life, and favorable safety profiles. All study protocols administered mepolizumab every four weeks as an add-on treatment to current asthma therapies [30, 33-35].

DREAM Trial

This 52-week multicenter, double-blind, placebo-controlled trial was conducted from November 9, 2009 through December 5, 2011 in eosinophilic-inflamed patients with a history of recurrent severe asthma exacerbations [33]. Six hundred and twenty-one patients were randomized into the mepolizumab group (receiving intravenous mepolizumab doses of 75 mg, 250 mg, or 750 mg) and placebo (receiving 100 mL of 0.9% sodium chloride). Findings from this study reported a rate of 2.4 patients per year experiencing clinically significant exacerbations within the placebo group, with the 75 mg, 250 mg, and 750 mg mepolizumab groups reporting rates of 1.24 (48% reduction, 95% CI 31 - 61%; p < 0.0001), 1.46 (39% reduction, 19 - 54%; p = 0.0005), and 1.15 (52% reduction, 36 - 64%; p < 0.0001) patients per year, respectively. In summary, the trial deemed mepolizumab to be effective and well-tolerated.

MENSA Trial 

MENSA was a randomized, double-blind, double-dummy study involving 576 patients with recurrent asthma exacerbations and eosinophilic inflammation despite high-dose inhaled glucocorticoid use [31]. Patients receiving intravenous mepolizumab demonstrated relative exacerbation rate reductions of 47% vs. placebo (95% CI, 28 - 60%), while those receiving subcutaneous mepolizumab reported relative reduction rates of 53% (95% CI, 36 - 65%, p < 0.001 for both comparisons). Furthermore, cases of exacerbations requiring either admission to a hospital or an emergency department visit were reduced by 32% within the intravenous mepolizumab group and 61% within the subcutaneous group. There were also average FEV_1_ increases of 100 mL and 198 mL in the intravenous and subcutaneous groups, respectively, when compared with the placebo group.

SIRIUS Trial 

The SIRIUS study was a randomized, double-blind trial consisting of 135 patients suffering from severe eosinophilic-type asthma [34]. The glucocorticoid-sparing effect of mepolizumab, 100 mg, as compared to placebo, were both administered subcutaneously once every four weeks for a duration of 20 weeks. Primary outcomes reported the likelihood of any reduction in glucocorticoid usage to be 2.39 times greater within the mepolizumab group than within the placebo group (95% CI 1.25 - 4.56; p = 0.04). Additionally, the mepolizumab group experienced a median 50% reduction from baseline glucocorticoid doses when compared to placebo (p = 0.007), as well as a 32% relative reduction in annualized rates of exacerbations (1.44 vs. 2.12, p = 0.04) and a 0.52-point reduction of asthma symptoms (p = 0.004) based on the Asthma Control Questionnaire (ACQ) (where a 0.5 point difference is considered clinically significant). The study concluded that in patients using oral glucocorticoids as maintenance therapy, the use of mepolizumab provided significant glucocorticoid-sparing effects, as well as reductions in asthma exacerbations and symptoms.

COSMOS Trial 

The COSMOS study consisted of patients from the previous clinical trials MENSA and SIRIUS and was performed as a 52-week open-label extension study [35]. Five hundred and fifty-eight patients (86%) reported experiencing adverse effects (358 from the mepolizumab groups and 200 from the placebo group), while 94 patients (14%) reported serious adverse events (58 previously on mepolizumab and 36 previously on placebo). Patients within the mepolizumab group reported no fatal adverse effects or anaphylaxis, 13 (2%) experienced systemic reactions, and 29 (4%) experienced localized site reactions. Overall, the data from this study displays that in patients with severe eosinophilic asthma, mepolizumab has a favorable safety profile, as well as a stable effect on long-term maintenance treatment [35].

Mepolizumab’s pharmacokinetic and pharmacodynamic information is found [32] in the Appendix, Table 1.

Benralizumab

Benralizumab (Faserna®, AstraZeneca, Cambridge, United Kingdom) is a fully-humanized, afucosylated anti-IL-5Rα monoclonal antibody that inhibits IL-5 receptor signaling and produces antibody-directed cell-mediated cytotoxicity of eosinophils and basophils [36]. Unlike reslizumab and mepolizumab which target the IL-5 cytokine, benralizumab targets the IL-5 receptor itself, blocking activation of eosinophils and enhancing antibody-dependent cytotoxicity. Benralizumab is indicated as an add-on treatment for severe eosinophilic asthma that is uncontrolled by inhaled corticosteroids in patients 12 years of age and older. However, benralizumab is unique from other IL-5 antagonists in that it not only depletes eosinophils from circulation but from the bone marrow, sputum, airways, and lungs as well [36]. Benralizumab binds specifically to isoleucine-61, found in domain 1 of the human IL-5Rα, thus interacting with the extracellular IL-5Rα epitope located near the IL-5 binding site. Benralizumab then inhibits heterodimerization of IL-5 receptor α/β subunits and the signal transduction pathway that follows. In doing so, the growth, differentiation, recruitment, activation, and survival of eosinophils is inhibited. The second mechanism of action of benralizumab is through binding of Fc fragments to the Fc𝚢IIIRa receptor, expressed by natural killer cells, macrophages, and neutrophils, thereby promoting apoptosis of eosinophils through the release of proapoptotic proteins [37].

Dosing for benralizumab as add-on maintenance treatment of severe asthma is a 30 mg subcutaneous injection every four weeks for the first three doses, then once every eight weeks thereafter. The injection comes as a single-dose prefilled syringe with a 30 mg/mL solution [38].

Patients with severe airway inflammation cannot always be adequately controlled by inhaled corticosteroids, and in some cases, they also cannot be controlled by systemic corticosteroids. Benralizumab’s dual mechanism of action gives a potentially superior efficacy profile to other agents of the same class; however, few trials have been completed to determine whether this is the case in vivo. A meta-analysis reviewing 10 trials comparing mepolizumab, reslizumab, and benralizumab did, however, determine that there were no significant differences between the three drugs’ safety and efficacy profiles when treating severe eosinophilic asthmatic patients [38]. In addition to this meta-analysis, three randomized, double-blind, parallel-group, placebo-controlled, multicenter studies (Efficacy and Safety Study of Benralizumab Added to High-dose Inhaled Corticosteroid, Plus LABA in Patients with Uncontrolled Asthma (SIROCCO), Efficacy and Safety Study of Benralizumab in Adults and Adolescents Inadequately Controlled on Inhaled Corticosteroid, Plus Long-acting β2 Agonist (CALIMA), and Efficacy and Safety Study of Benralizumab to Reduce OCS Use in Patients with Uncontrolled Asthma on High-dose Inhaled Corticosteroid, Plus LABA and Chronic OCS Therapy (ZONDA)) [39-41] were completed to test the efficacy and safety of benralizumab prior to its release, along with one Phase 2, randomized, controlled, dose-increase study [42].

SIROCCO Trial 

The SIROCCO trial examined the efficacy and safety of benralizumab in patients with severe asthma uncontrolled by concomitant high-dose inhaled corticosteroids and long-acting β-2 agonists [39]. In this trial, 1,205 patients were divided into control and experimental arms. The usual asthma therapy for both groups was a high-dose inhaled corticosteroid and a long-acting β-2 agonist for at least one year prior to the start of the study. The experimental group added benralizumab, 30 mg every four weeks or every eight weeks, to the usual therapy, while the control group used a placebo at similar intervals. The SIROCCO trial continued for 18 months; endpoints included the number of annual asthma exacerbations, prebronchodilator FEV_1_, asthma symptoms, and adverse events. Benralizumab, given either every four weeks or every eight weeks, reduced the annual asthma exacerbation rates (both with p < 0.0001), as well as prebronchodilator FEV_1_. Overall asthma symptom improvements, however, were only seen within the eight-week regimen of benralizumab. The most common adverse events that occurred in patients taking benralizumab were worsening asthma and nasopharyngitis [39].

CALIMA Trial 

The CALIMA trial examined the efficacy and safety of benralizumab in patients with severe, uncontrolled, eosinophilic asthma as an add-on therapy to high-dose inhaled corticosteroids and a long-acting β-2 agonist, with a history of two or more asthma exacerbations in the previous year [40]. In this trial, 1,306 patients were divided into one control arm and two experimental arms. The experimental groups received benralizumab, 30 mg every four weeks and every eight weeks, while the control group received a placebo in addition to their usual asthma therapy. The CALIMA trial continued for a total of 19 months, with measured endpoints including asthma exacerbation rates, prebronchodilator FEV_1_, asthma score, and adverse events. Benralizumab lowered the annual asthma exacerbation rates in both the four-week (p = 0.0018) and eight-week (p = 0.0188) regimens when compared to placebo. Additionally, benralizumab significantly improved prebronchodilator FEV_1_ for patients in both the four-week and eight-week groups over placebo. However, the total asthma symptom score was reduced only within the eight-week regimen. The most common adverse effects in patients taking benralizumab were worsening asthma and nasopharyngitis [40].

ZONDA Trial 

The ZONDA trial examined whether benralizumab was as effective as oral glucocorticoid-sparing therapy in patients relying on oral glucocorticoids for the management of their severe eosinophilic asthma [41]. In this trial, 220 patients randomly received either benralizumab, 30 mg every four weeks, every eight weeks, or a placebo. This trial lasted for seven months and the primary endpoint was the percentage change in the oral glucocorticoid dose from baseline to Week 28. Asthma exacerbation rates, lung function, asthma symptoms, and adverse effects were also assessed. Both the four-week and eight-week regimens significantly reduced the final oral glucocorticoid doses from baseline by 75% vs. placebo (p < 0.001 for both). Furthermore, asthma exacerbation rates were lower in patients treated with benralizumab by 55% within the four-week regimen (p = 0.003) and 70% within the eight-week regimen (p < 0.001). There were no differences between the FEV_1_ in the patients treated with benralizumab when compared with placebo over the 28 weeks. Asthma symptoms varied widely across all patients observed; thus, differences between groups were not found to be statistically significant. The number of adverse events between both groups were comparable [41].

Phase 2

The Phase 2 trial observed the reductions in eosinophil biomarkers by benralizumab in patients with eosinophilic asthma. Blood serum was collected from patients who received benralizumab treatment (n = 24) and healthy volunteers (n = 20). Eosinophilic biomarkers were measured in all patients prior to the administration of benralizumab and once more after treatment was completed. The biomarkers measured were as follows: blood eosinophils, IL-5, eosinophil-derived neurotoxin (EDN), eosinophil cationic protein (ECP), eotaxin/chemokine (C-C motif) 11 (CCL11), eotaxin-2/CCL24, tumor necrosis factor (TNF), and interferon-γ (IFN-γ). There were no changes in serum TNF or IFN-γ in patients taking benralizumab when compared to placebo. Of the measured biomarkers, increases were seen in EDN concentrations, IL-5, and eotaxin concentrations (2/CCL24, CCL11) in patients taking benralizumab [42].

Benralizumab’s pharmacokinetics and pharmacodynamics information [38] can be found in the Appendix, Table 1.

Dupilumab

Dupilumab (Dupixent®, Sanofi Biotechnology, Bridgewater, NJ, USA) is a fully-humanized IgG4 monoclonal antibody which binds to the α subunit of IL-4 receptors, inhibiting the signaling of both IL-4 and IL-13 [43-46]. These two interleukins are secreted mainly by CD4+ Type 2 helper T-cells and are responsible for the following [43]: • Class switching of IgM to IgE which activates the inflammation cascade (mast cells, eosinophils) • Stimulation of Janus kinase and tyrosine kinase proteins, leading to signal transduction and transcription activation • Airway remodeling through collagen accumulation, smooth muscle proliferation, hyperplasia of goblet cells, and conversion of fibroblasts to myofibroblasts • Increased airway smooth muscle contraction • Increased mucus production

Because these pro-inflammatory changes lead to increases in hyperresponsiveness of the airway, blocking these actions can help in slowing or ceasing progression of the disease and reversing airway obstruction. The results of the human trials demonstrated mixed results. IL-4 and IL-13 were comparable in many respects. IL-4 aids in directing naïve CD4+ Th cells towards a Th2 phenotype, while IL-13 enhances airway inflammation and remodeling [43]; dupilumab has dual inhibition of both IL-4 and IL-13 which demonstrates more beneficial effects in asthma therapy.

Dupilumab received FDA approval in October 2018 for the indication of add-on therapy for asthma in patients 12 years and older with moderate to severe persistent asthma, as an adjunct to inhaled corticosteroids and long-acting β-2 agonists [43-47]. Dupilumab dosing consists of a loading dose of 600 mg or 400 mg (for ages 12 - 17 weight < 60 kg) to be injected subcutaneously in divided doses, with a maintenance dose of 300 mg or 200 mg (for ages 12 - 17 weight < 60 kg) injected subcutaneously every other week. Thus far, there are no recommendations for dose adjustments in renal or hepatic insufficiency [48].

Dupilumab Trial I

Trial I was a randomized study that recruited a total of 104 patients (52 in the dupilumab group, 52 in the placebo group) with moderate to severe asthma and blood eosinophil levels of at least 300 cells per microliter over a 12-week period. Study patients were on both inhaled corticosteroid and long-acting beta-agonist (LABA) therapies. The primary outcome measured asthma exacerbations for each arm of the study. In the dupilumab group, three patients developed exacerbations, while 23 exacerbations occurred within the placebo group [44].

Secondary outcomes measured were the time since last asthma exacerbation and lung function change from baseline (including FEV_1_, morning and evening peak expiratory flow, ACQ score, symptomatic nocturnal awakenings, and the number of rescue inhalations used per day). For all secondary outcomes, small improvements from baseline were noted within the dupilumab group, which stabilized after two weeks, while the placebo group experienced either worsening of symptoms or no change. Inflammatory markers associated with Th2, such as the fraction of exhaled nitric oxide (FENO), serum biomarkers, plasma exotoxin-3, and peripheral blood eosinophil levels, were reduced in the dupilumab group (p-value < 0.001), except for eosinophil levels, where no change was noted with either group. Adverse events reported in the study included injection site reactions, pharyngitis, nausea, and headache (81% in the dupilumab group, 77% in the placebo group). No deaths occurred in either of the groups during the study [44].

Dupilumab Trial II

Trial II was a multicenter randomized study that recruited 769 patients (611 in the dupilumab groups, 158 in the placebo) with moderate to severe asthma and using both inhaled corticosteroid and LABA therapies. The study compared four different dosing regimens of dupilumab (200 mg subcutaneously every two weeks, 200 mg subcutaneously every four weeks, 300 mg subcutaneously every two weeks, and 300 mg subcutaneously every four weeks), further stratifying patients by blood eosinophil counts of at least 300 cells/mcL and patients with eosinophil counts less than 300 cells/mcL [45].

The primary outcome measured was the change in FEV_1_ from baseline to Week 12. In the group with eosinophil counts ≥ 300 cells/mcL, the highest increase in FEV_1_ was reported in two dosing arms: 200 mg every two weeks (Q2W) (p = 0.0008) and 300 mg Q2W (p = 0.0063). In the < 300 cells/mcL group, similar results were found in two dosing arms: 200 mg Q2W (p = 0.0043) and 300 mg Q2W (p = 0.0086). Secondary outcomes in the trial were exacerbation rates, time of exacerbation occurrence, and associated asthma symptoms. All results in the placebo group were statistically insignificant. All dupilumab dosing groups experienced a significant reduction in exacerbation rates, with the greatest reductions occurring in the 200 mg Q2W and 300 mg Q2W dosing arms. Exacerbations were also notably delayed in the dupilumab groups, regardless of eosinophil count [45].

Adverse events reported in the study included upper respiratory tract infection, injection site erythema, and headaches. These rates occurred at similar rates across all groups (75% in placebo, 75 - 83% in dupilumab). Serious adverse event rates were 6% in the placebo group, while it was 7% in the combined dupilumab regimen. Rates of serious events requiring discontinuation of the drug were 3% in the placebo group and approximately 4% in the combined dupilumab regimen. Two patients died within the dupilumab (300 mg Q4W) arm - one due to acute cardiac failure and the other due to many factors, such as metastatic gastric cancer, pneumonia, and pulmonary heart disease [45].

Dupilumab’s pharmacokinetic and pharmacodynamic information [48] is found in the Appendix, Table 1.

## Conclusions

Clinical trials evaluating the efficacy of new biologic agents, such as omalizumab (Xolair®), reslizumab (Cinqair®, US, Cinqaero®, EU), mepolizumab (Nucala®), benralizumab (Faserna®), and dupilumab (Dupixent®), have shown significant relative reductions in exacerbation rates, increases in FEV_1_, and occasionally reductions in inflammatory markers when used in patients with refractory asthma despite maximal usage of available β2-agonists and/or inhaled corticosteroids. Guidelines will be essential in determining which category of patients may receive each of these agents. Biomarkers will also come into play in aiding clinicians in making such determinations. While there is promise in the treatment of eosinophilic-mediated severe asthma, further research is warranted for the development of therapies focused on non-eosinophilic inflammatory pathways, as well as those with low Th2 expression.

## Appendices

**Table 1:** Drug Properties DNA: deoxyribonucleic acid; EU: Europe; US: United States; Vd: Volume of Distribution

DRUG PROPERTIES				
	Drug Type	Mechanism of Action	Pharmacokinetics	Pharmacodynamics
Omalizumab (Xolair®)	Recombinant humanized DNA-derived IgG1 monoclonal antibody	Binds with high affinity to the heavy chain C-epsilon-3 (C-ε-3) domain of IgE, thereby preventing binding and activation of basophils and mast cells.	Absorption: Slow absorption from subcutaneous depot; Distribution: Vd = 78 ± 32 mL/kg; Metabolism: endothelial cells in the liver degrade both IgG:IgE receptor complexes and omalizumab:IgE receptor complexes; Excretion: primarily hepatic degradation, some intact IgG (and potentially omalizumab) is thought to be excreted in bile.	Clearance: 2.4 ± 1.1 mL × kg−1 × day−1 based on population kinetics. A doubling of body weight doubles the apparent clearance of the drug. Onset: unknown. Typically, patients demonstrate a measurable response between 12 and 16 weeks of treatment; Time to peak: 7 - 8 days; Elimination half-life: 24 to 26 days; Bioavailability: 62%
Reslizumab (Cinqair®, US, Cinqaero®, EU)	Immune globulin G4-kappa-type (IgG4 κ) monoclonal antibody	Impairs bioactivity of IL-5 by blocking its binding ability to the IL-5R α-chain complex expressed on the surface of eosinophils, thereby inhibiting IL-5 signaling and reducing both the production and survival of eosinophils.	Absorption: rapid; Distribution: Vd = 5 L; Metabolism: enzymatic proteolysis into small peptides and amino acids; Excretion: unknown (non-renal)	Clearance: 7 mL/hr. Onset: Unknown – eosinophil count reduction was noted within 2 to 3 days after first dose. Time to peak: 6.9 hours. Elimination half-life: 25 to 30 days. Bioavailability: Approx. 67%.
Mepolizumab (Nucala®)	IgG1κ monoclonal antibody	Impairs the bioactivity of IL-5 by blocking its binding ability to the IL-5 α-chain complex, inhibiting eosinophil production and survival.	Absorption: subcutaneous; Distribution: Vd ~3.6 L; Metabolism: proteolytic degradation through enzymes found throughout the body (including hepatic tissue); Excretion: unknown (non-renal)	Clearance: ~0.28 L/day for a 70 kg patient; Onset: unknown; Time to peak: unknown; Elimination half-life: 16 to 22 days; Bioavailability: approx. 80%
Benralizumab (Faserna®)	Fully humanized afucosylated anti-IL-5Rα monoclonal antibody	Inhibits IL-5 receptor signaling and produces antibody-directed cell-mediated cytotoxicity of eosinophils and basophils	Absorption: subcutaneous administration; Distribution: Vd = 3.2 L (central), 2.5 L (peripheral) for a 70 kg individual; Metabolism: undergoes proteolytic degradation via enzymes widely distributed in the body; Excretion: linear; Pharmacokinetics: no evidence of target receptor-mediated clearance	Clearance: 0.29 L/day for a 70 kg individual; Elimination half-life: approximately 15 days; Bioavailability: approximately 58%
Dupilumab (Dupixent®)	Fully humanized IgG4 monoclonal antibody	Binds to the α subunit of IL-4 receptors, inhibiting the signaling of both IL-4 and IL-13. These two interleukins are secreted mainly by CD4+ Type 2 helper T-cells	Absorption: subcutaneous, non-linear; Distribution: Vd = 4.8 ± 1.3 L; Metabolism: via monoclonal antibody receptor complex internalization and degradation of monoclonal antibody into smaller polypeptides and amino acids (catabolism); Excretion: unknown. Median time to undetectable levels is 9 - 13 weeks, depending on dosing regimen	Clearance: linear, 0.126 L/day; Time to peak: approx. 7 days; Elimination half-life: approx. 26 days; Bioavailability: 60.7%

**Table 2:** Summary of Trial Data ACT: Asthma Control Test; COSMOS: A Multi-centre, Open-label, Long-term Safety Study of Mepolizumab in Asthmatic Subjects Who Participated in the MEA115588 or MEA115575 Trials; DREAM: Dose-ranging Efficacy and Safety with Mepolizumab in Severe Asthma; ED: emergency department; FEV_1_: forced expiratory volume in 1 second; ICS: inhaled corticosteroids; IV: intravenous; LABAs: long-acting β2 agonists; MENSA; Mepolizumab as Adjunctive Therapy in Patients with Severe Asthma; OCS: oral corticosteroids; SIRIUS: Mepolizumab Steroid-sparing Study in Subjects with Severe Refractory Asthma; SIROCCO: Efficacy and Safety Study of Benralizumab Added to High-dose Inhaled Corticosteroid, Plus LABA, in Patients with Uncontrolled Asthma; SQ: subcutaneous; ZONDA: Efficacy and Safety Study of Benralizumab to Reduce OCS Use in Patients with Uncontrolled Asthma on High-dose Inhaled Corticosteroid, Plus LABA and Chronic OCS Therapy

SUMMARY OF TRIAL DATA		
BIOLOGIC	RESEARCH TRIAL	SUMMARY OF PRIMARY OUTCOME
Omalizumab	Trial I	ICS, plus LABAs, plus one or more additional asthma controller medications, excluding OCS; and M3 was ICS, plus LABAs, plus OCS. The omalizumab group experienced a 25% relative reduction in asthma exacerbation rates vs. placebo over a 48-week study period (0.66 vs. 0.88; p = 0.006).
	Trial II	Patients with poorly controlled asthma at baseline (defined as an ACT score ≤ 15), saw significant benefits (ACT score increases) with Omalizumab but only after 24 weeks
	Trial III	Omalizumab was associated with a 37% relative risk reduction vs placebo in asthma exacerbations (0.146 vs. 0.233, p = 0.009).
Reslizumab	Study I/II	The reslizumab group had lower rates of total asthma exacerbations compared to those in the placebo group, with observed relative reductions of 50% in Study I, and 41% in Study II. The studies also demonstrated relative reductions of 66% in Study I and 69% in Study II for exacerbations requiring admission to hospital or ED visits.
	Study III	This study observed severe asthma patients with at least 400 eosinophils per mcl. Patients on 3 mg/kg reslizumab experienced an increase in FEV_1_ 0.16 L greater than placebo. In the reslizumab 0.3 mg/kg group an FEV_1_ increase 0.116 L greater than placebo was observed.
	Study IV	The study observed moderate-to-severe eosinophilic asthma patients, the majority of whom had eosinophil counts < 400 per mcl. Patients on 3 mg/kg of reslizumab experienced an FEV_1_ change 0.076 L greater than placebo.
Mepolizumab	DREAM Trial	In eosinophilic-inflamed patients with a history of recurrent severe asthma exacerbations, mepolizumab doses ranging from 75 mg to 750 mg, showed relative reductions of 39% to 52% in yearly exacerbations.
	MENSA Trial	Patients receiving IV mepolizumab demonstrated relative exacerbation rate reductions of 47% vs. placebo, while those receiving SQ mepolizumab reported reduction rates of 53%.
	SIRIUS Trial	The use of mepolizumab provided significant glucocorticoid-sparing effects (median 50% reduction from baseline glucocorticoid dose), as well as reductions in asthma exacerbations and symptoms.
	COSMOS Trial	This was an open-label extension study evaluating patients from MENSA and SIRIUS trials. In patients with severe eosinophilic asthma, mepolizumab showed a favorable safety profile and stable effect on long-term maintenance treatment.
Benralizumab	SIROCCO Trial	Overall, benralizumab saw reductions in annual asthma exacerbation rates and improvements in FEV_1_. However, only those receiving benralizumab every 8 weeks (instead of 4 weeks) showed improvements in asthma symptoms.
	CALIMA Trial	Overall, benralizumab saw reductions in annual asthma exacerbation rates and improvements in FEV_1_. However, the total asthma symptom score was reduced only in the 8-week regimen.
	ZONDA Trial	Both the 4-week and 8-week benralizumab regimens significantly reduced the median oral glucocorticoid doses from baseline by 75% vs. placebo 25%.
Dupilumab	TRIAL I	Dupilumab was associated with an 87% relative reduction in numbers of exacerbations vs. placebo.
	TRIAL II	The primary outcome measured was the change in FEV_1_ from baseline to week 12. Dupilumab was associated with an overall significant increase in FEV_1_ (all dosing regimens showed this trend, except 200 mg every 4-week dosing.)
